# Spirituality and religiousness as predictors of life satisfaction among Peruvian citizens during the COVID-19 pandemic

**DOI:** 10.1016/j.heliyon.2021.e06939

**Published:** 2021-05-05

**Authors:** Renzo Felipe Carranza Esteban, Josue Edison Turpo-Chaparro, Oscar Mamani-Benito, Jesús Hanco Torres, Fiorella Sarria Arenaza

**Affiliations:** a Universidad San Ignacio de Loyola, Fontana 550, La Molina, 15024, Lima, Peru; b Escuela de Posgrado, Universidad Peruana Unión, Carretera Central Km 19.5 Ñaña, Chosica, Lima 15, Lima, Peru; c Universidad Nacional de San Agustín de Arequipa, Calle Santa Catalina 117, Arequipa, Peru

**Keywords:** Religiousness, Spirituality, Life satisfaction, Predictive analysis, Peruvian citizens

## Abstract

The objective of this study was to determine if religiousness and spirituality predict life satisfaction among Peruvian citizens during the COVID-19 pandemic. This is a non-experimental, predictive and cross-sectional study with a sample of 734 people of both sexes (39.5% males and 60.5% females) between 17-75 years of age (M = 32.05). To measure the variables, the Brief Multidimensional Measure of Religiousness/Spirituality (BMMRS) and the Satisfaction with Life Scale (SWLS) were used. A multiple regression analysis was performed to determine the variables that best predict life satisfaction, finding that the spirituality variable explains 10.7 % of the total variance of the life satisfaction variable. In summary, a positive and significant correlation between spirituality and life satisfaction is identified (r = .328, p < .01).

## 1 Introduction

The World Health Organization (WHO) declared the outbreak of the new coronavirus (COVID-19) a public health emergency on January 2020 and on March 11 2020, this organization declared it a pandemic (Ruiz-Manriquez et al., 2020).

From that point on, the repercussions were evident not only in terms of physical health, but also in aspects such as lifestyle, personal habits and people's religious beliefs, in large part due to the measures of social isolation and restrictions imposed in almost every country in the world (Ghosh et al., 2020).

Although COVID-19 is obviously important from a biological point of view, psychological and social factors that influence people's behaviors have turned the situation into a world disaster, one of the worst health phenomena in recent years (Urzúa et al., 2020). In this context, some researchers such as Li et al. (2020) have explored its impact on mental health among the general population, finding an increase in anxiety, depression and other feelings such as indignation, frustration and feelings of social risk as well as a decrease in positive emotions (happiness) and life satisfaction; the latter, considered one of the most important constructs for evaluating subjective well-being (Caycho-Rodríguez et al., 2018d). Specifically, satisfaction with life refers to a cognitive judgment process in which a person is evaluated according to their own criteria or standards (Gutiérrez et al., 2014), that is, an assessment including various aspects of the person's life such as family, studies, work, health, friends and free time (Schnettler et al., 2014).

As found in the relevant literature, many factors promote and inhibit life satisfaction; of particular note for this study, people's religiosity and spirituality play an important role since they are considered psychosocial resources positively associated with psychological well-being (Barreto et al., 2015) and a better mental health condition (Jafari et al., 2010). In general, there is ample acceptance by the scientific community that religion should be classified as one of the determining factors of a person's health, even more so in the current context, where spiritual and religious beliefs in patients with COVID-19 have been shown to help foster mental relaxation and calm (Fardin, 2020).

Religiosity is understood as a social and objective experience with a superior being, framed in practices carried out by people affiliated with a religious organization who maintain a set of beliefs, values and doctrines (Koenig, 2004). Here, it is important to make a distinction with spirituality, which is an individual, interior and subjective experience that transcends the biological, the psychological and the social (Rivera-Ledesma and Montero, 2005). Although historically they were considered a single construct, the studies of Johnstone et al. (2009) showed the need to divide them. Therefore, spirituality must be understood as a human condition in which a meaning and purpose is sought in a higher being, while religiosity is framed in the practice of activities of the religious community to which one adheres, respecting its doctrines, rites and norms (Fonseca Canteros, 2016).

Regarding the relationship between these variables, the scientific literature reports studies where religiosity has been shown to have a significant influence on psychological functioning (Przepiorka and Sobol-Kwapinska, 2018). Thus, a study published in 2017 evaluated the moderating effect of religious beliefs in the relationship between economic income and life satisfaction, finding a positive effect at the individual level, yet a negative effect at the country level (Plouffe and Tremblay, 2017). Another study published in 2019 that included a Canadian immigrant population 15 years of age or older, found a negative effect of religious discrimination on life satisfaction, on the other hand, higher religiosity was associated with higher levels of satisfaction (Vang et al., 2019). This result is very similar to that found in 2012, when Park et al. found that religious commitment is an important factor in improving the quality of life of elderly Korean immigrants (2012). However, despite these evidences there are also studies that contradict these findings; a clear example of this is a study published in 2019 that collected information from 59 countries between the years 2010–2014, which tested two patterns found by Plouffe and Tremblay (2017) who concluded that in most countries, the rich are happier than the poor and those who believe in God are happier than non-believers. In this case, when they reanalyzed the data using other types of measures, they found that religiosity does not have a robust effect on life satisfaction, although they recognize that in most countries religious people are happier than the average (Bomhoff and Siah, 2019).

Regarding spirituality and satisfaction with life, these variables are considered psychological factors related to behaviors that promote health (Zadworna-Cieślak, 2020). As a result, it can be assumed that there is a causal relationship between them, as revealed by some studies such as the one published in 2011, which took Korean elderly people as the population, finding that the perception of spiritual well-being was directly associated with the perception of high satisfaction with life (Lee, 2011). Another similar population study revealed in 2020 that, after examining the elderly aged 60 to 99 in Poland, life satisfaction plays a mediating role in the relationship between spirituality and health behavior (Zadworna-Cieślak, 2020). On the other hand, studies in younger populations such as industrial employees in Indonesia, found that Islamic religiosity has a significantly positive impact on job satisfaction in small and medium-sized companies in the embroidery industry (Amaliah et al., 2015). Similarly, in a study carried out with infertile women from Iran (Etemadifar et al., 2016), in a population of athletes from the national team of the same country (Mirzaaghazadeh et al., 2016), and in professionals from the anthroposophic health field in Switzerland (Büssing et al., 2015), the same effect was demonstrated.

In summary, most of the studies carried out on the subject account for the positive effects of variables such as spirituality, religiosity, spiritual well-being, religious beliefs, among others, on variables of the psychological field, especially satisfaction with life (Javanmard, 2013). In such a scenario, religiousness and spirituality have been much more studied in health contexts, elderly people (Zimmer et al., 2016) and the palliative treatment of terminally ill patients like patients with cancer (Ahmadi et al., 2015); therefore, it is important to take into account that a perspective on spiritual life can have an effect on optimism and people's levels of satisfaction (Salmani et al., 2020).

Taking this literature review into account, the present study proposes as a hypothesis that spirituality and religiosity predict satisfaction with life in Peruvian adults during the COVID-19 pandemic. Thus this hypothesis needs to be tested, taking into account the context of the health emergency that exists in Peru, a country with a high percentage of religious population, and, considered the epicenter of the COVID-19 Pandemic in Latin America and the world (BBC, 2020), due to the high rates of infection and deaths in 2020 and so far in 2021.

For all the aforementioned, the objective of this study was to determine if religiousness and spirituality predict life satisfaction among Peruvian citizens during the COVID-19 pandemic.

## 2 Material and method

This is a non-experimental, predictive study (Ato, López-García, & Benavente, 2013) that considers life satisfaction as its criterion variable and spirituality and religiousness as predictive variables.

### 2.1 Participants

A non-probabilistic sampling was performed. From a total of 734 Peruvian citizens that participated in the study, 290 were men (39.5%) and 444 were women (60.5%), with ages between 17-75 years old (M = 32.05; DE = 14.63).

With regard to the main sociodemographic characteristics, 68.5% people have a higher education degree, 72.9% are Adventist and 21% express that they have been following this religion for 31 years or more.

### 2.2 Instruments

The Brief Multidimensional Measure of Religiousness/Spirituality (BMMRS; Fetzer Institute and National Institute of Aging Working Group, 1999), validated in Spanish by Gallardo-Peralta, Cuadra-Peralta & Veloso-Besio (2018) was used to measure religiousness and spirituality. The instrument consists of 16 items of which eight evaluate religiousness and the others evaluate spirituality. For the study, BMMRS reported an adequate reliability (α = .78 [CI95%: .75 - .80]).

The Satisfaction with Life Scale (SWLS; Diener et al., 1985) was also used, which was translated and validated in Spanish by Atienza et al. (2000). Additionally, it was adapted to the Peruvian context by Caycho-Rodríguez et al. (2018d). This is a brief measure composed of five items that evaluate the level of a person's life satisfaction. It is a Likert-type scale format with 5 response options which indicate: (1) “totally disagree” to (5) “totally agree”. In this study, the reliability of the SWLS was: α = .76 (CI95%: .72 - .78).

### 2.3 Method

This research study was carried out within the context of the health emergency declared by the Peruvian government. An online form was developed using Google Forms, which was shared via email and through social networks. Before the participants completed the questionnaire, an informed consent statement was presented stating the objectives of the study, the voluntary and anonymous nature of their participation, and also informing that the information collected was only to be used for research purposes.

### 2.4 Statistical analysis

First, a descriptive analysis of the variables including life satisfaction, religiousness and spirituality was carried out. Second, differences between variables were analyzed by sex. Student's t test and Cohen's d were used to measure the effect size (ES) in the comparison between the two independent groups (Caycho-Rodríguez, 2018a), where values of .20 .50 and .80 express a small, moderate and large ES, respectively (Cohen, 1998; Ferguson, 2009). The correlation analysis was carried out using Pearson's coefficient according to Ferguson's proposal (2009) (r ≥ .50: large, r ≥ .30: average and r ≥ .10: small). Finally, a regression model was estimated, calculating the ES depending on the coefficient of determination (R2) and its confidence intervals, where ≥0.02, ≥0.13 and ≥0.26 values indicate small, average, and large ES, respectively (Caycho-Rodríguez, 2018b; (Caycho-Rodríguez, 2018c). SPSS version 24.0 statistical software was used for statistical analyses.

### 2.5 Ethical approval

The study was approved by the ethics committee of the Universidad Peruana Union number 2020-CEUPeU-00016. Participation was voluntary and informed consent was obtained from all participants.

## 3 Results

### 3.1 Descriptive analyses

Table 1 shows descriptive analyses of the study variables, and it is worth noting that the coefficients of asymmetry and coefficients of kurtosis are usually below 1.5 (Pérez & Medrano, 2010).

**Table 1:** Descriptive analysis of life satisfaction, spirituality and religiousness.

Variables	M	SD	A	K
Life satisfaction	17,77	3,555	-,472	,340
Spirituality	33,79	5,190	-,1,237	2,142
Religiousness	32,24	5,924	-,1,166	,922

Note: M = mean; SD = standard deviation; A = coefficient of asymmetry; K = coefficient of kurtosis.

### 3.2 Differences between life satisfaction, spirituality and religiousness according to sex

Student's t test for independent samples (Table 2) indicates that there are no significant differences between life satisfaction, spirituality and religiousness among males and females (p > 0.05). The effect size calculation for Cohen's d was < = .20.

**Table 2:** Life satisfaction, spirituality and religiousness among males and females.

	Males		Females		t	p	*d*
	M	SD	M	SD			
Life satisfaction	17,84	3,947	17,72	3,277	,428	0,669	0.09
Spirituality	33,31	5,318	34,10	5,087	-2,011	0.045	0.15
Religiousness	31,74	6,303	32,57	5,646	-1,850	0.065	0.14

### 3.3 Correlation between life satisfaction, spirituality and religiousness

Table 3 shows the correlation coefficients between life satisfaction, spirituality and religiousness where a direct and significant correlation between the study variables is observed (p < .01).

**Table 3:** Correlation between life satisfaction, spirituality and religiousness.

	Life satisfaction	Spirituality	Religiousness
Life satisfaction	1		
Spirituality	,328∗∗	1	
Religiousness	,132∗∗	,541∗∗	1

∗∗ Significant at the 0,01 level (bilateral).

### 3.4 Regression analysis of spirituality and religiousness as predictors of life satisfaction

A multiple regression analysis was performed to determine the variables that best predict life satisfaction; spirituality and religiousness were included, where spirituality was identified as the most predictive of the two. Table 4 shows multiple correlation coefficients R, R2, R2-corrected, standard error estimation (SE), and ANOVA F-value.

**Table 4:** Multiple correlation coefficients R, R2, R2-corrected, SE, F.

Model	R	R2	R2-corrected	SE	F	Sig
1	,328^a^	,107	,106	3,361	88,103	0,000^b^

a Predictive variable: (constant), Spirituality.

b Dependent variable: Life satisfaction.

As shown in Table 4, the determination coefficient R2 = .107 indicates that spirituality explains 10.7 % of the total variance of the criterion variable (life satisfaction). As the largest value of the multiple coefficient of determination, this variable explains more of the regression equation and consequently, has a greater value of predictive power for the dependent variable. R2-corrected explains 10.6 %. ANOVA F-value (F = 88.103, p = .000) indicates that there is a significant linear relationship between the predictive variable (spirituality) and the criterion variable (life satisfaction).

Table 5 shows non-standarized regression coefficients (B), standarized regression coefficients (β) and statistical coefficients related to the predictive variable. β coefficient (.429) indicates that spirituality (predictive variable) significantly predicts life satisfaction (criterion variable). The T value of beta regression coefficients of the predictive variable is highly significant (p < 0.01).

**Table 5:** Multiple regression coefficients (B) (non-standarized), β (standarized) and t Test.

Model	B	SE	β	t	Sig
1(Constant)	10,184	,818		12,457	,000
Spirituality	,224	,024	,328	9,386	,000

## 4 Discussion

Spirituality and religiousness have become an important resource for health improvement and population wellbeing during the COVID-19 pandemic (Koenig, 2020); different research studies point out that beliefs and religious practices help people to cope with stressful events, reduce anxiety and increase hope and life satisfaction (Park et al., 2012; Salmani et al., 2020). In that sense, the aim of this research study was to analyze if religiousness and spirituality predict life satisfaction among Peruvian citizens during the COVID-19 pandemic.

The results of the Student's t test indicated that there were no significant differences between life satisfaction, spirituality and religiousness among males and females (p > 0.05) for our sample. Previous studies reported similar results in relation to life satisfaction (Gutiérrez et al., 2014). In other studies, in the case of men, spirituality is related to better quality of life and health (Zavala et al., 2009). Additionally, other studies emphasize the importance of conducting additional studies on spirituality and gender roles in adult populations (Zadworna-Cieślak, 2020). Regarding magnitude of the effect, the evidence shows that there are no respective changes (< =.20); consequently, this finding may be in part because both men and women are similarly exposed to the COVID-19 pandemic (Castellanos-Torres et al., 2020) although a greater risk of mortality has been observed in men (Elgendy and Pepine, 2020). However, some studies on spirituality by gender report that the differences between them are significant (Kent, 2020; Rożnowski and Zarzycka, 2020).

Correlation analyses between life satisfaction, spirituality and religiousness demonstrate that there is a direct and significant association among them (p < .01). Spirituality and religiousness increased life satisfaction. This agrees with similar studies that report a significant and moderate relationship between life satisfaction and spirituality (Büssing et al., 2015). In the case of life satisfaction and religiousness, our results show a significant moderate relationship which is in line with similar studies that demonstrate that religious commitment is an important factor to improve life satisfaction (Park et al., 2012). Additionally, following religious norms, (Smith, 2003), as a part of religiousness improves social links, thus increasing life satisfaction. In this regard, people with high levels of spirituality are more likely to experience life satisfaction. This positive role has been studied and documented by several groups (Biccheri et al., 2016; Bovero et al., 2016).

On the other hand, although life satisfaction is related to a philosophy of life that takes into account health, communication and free time (Schnettler et al, 2014, 2017), spirituality is one of the most important factors that help people cope with stressful situations (Moreira-Almeida et al., 2014), which is an essential component in a person's life (Puchalski et al., 2014). In contrast, various researchers consider personality psychology a natural fit for the study of religion and spirituality (Kirkpatrick, 1999) or that the theory of personality is directly related to theology (Emmons and Paloutzian, 2003).

As many variables can have an impact on life satisfaction among Peruvian citizens, a regression analysis was carried out to identify significant predictors. The results of life satisfaction can be best explained by the spirituality variable, which explains 10.7 % of the variance. These results confirm the relationship between spirituality and life satisfaction, identifying that spirituality among members of a group is related to higher levels of life satisfaction. This is in line with the results of some research studies that have demonstrated that spirituality actions are directly related to the response to life satisfaction (Lee, 2011; Zadworna-Cieślak, 2020). Furthermore, previous studies (Wu et al., 2020), have pointed out that the positive practice of spirituality will bring about positive responses to life satisfaction, which implies a connection between these two factors (Koening, 2004; Maier and Surzykiewicz, 2020).

### 4.1 Theoretical implications

This study contributes to the literature related to the effects of religiosity and spirituality in what some have called the psychology of religion (Fonseca Canteros, 2016; Kirkpatrick, 1999; Przepiorka and Sobol-Kwapinska, 2018). While previous research considers religiosity and spirituality in a variety of behavioral intentions (Ahmadi et al., 2015; Barreto et al., 2015) our study focuses on their contributions to the satisfaction with life of Peruvian citizens in times of COVID-19. This study showed the significant effect of spirituality and religiosity on life satisfaction, a finding that is worth noting. This research also contributes to the literature on the effects of spirituality in times of a pandemic (Mejia et al., 2020; Quadri, 2020; Wildman et al., 2020). Unlike previous studies that consider the religiosity factor as an important predictor of well-being (Abbott, 2020; Emmons and Paloutzian, 2003), our study shows the preponderant role of spirituality in life satisfaction and that the spirituality of Peruvian citizens contributes to overall life satisfaction. Likewise, the results demonstrate the impact of spirituality on life satisfaction in a unique conceptual model. Although there are different studies that reveal the positive impact of religiosity and spirituality on life satisfaction, it is not common to carry out these studies in times of a pandemic. Using two independent questionnaires, this study demonstrated how spirituality experiences contribute to life satisfaction for Peruvian citizens while experiencing the weight of the COVID-19 pandemic. Finally, this study, as far as we know, is the first to evaluate these constructs in a Peruvian population in times of COVID-19.

### 4.2 Practical contributions

In regards to the practical contributions, it is important to analyze the Peruvian context. Peru is a country with a strong religious heritage and a particular Andean cosmology (Hill, 2010), considered a secular state, but which still maintains its heritage as a Catholic confessional state (Sánchez-Lasheras, 2016), where ethnic differences and the subordination of gender affects health interventions, and where women have been particularly affected as a vulnerable population (Espinosa, 2009). However, recent studies show that being religious, regardless of gender, can lead to certain conditions that affect mental health (Mejia et al., 2020). Our results confirm that the COVID-19 pandemic has affected everyone regardless of gender and despite the fact that women show higher levels of emotional instability (Rodríguez-Ramos et al., 2019), and that, in the case of Peru, religiosity can still have an impact on therapeutic decisions in both genders (Lavado Landeo, 2018).

On the other hand, spirituality is related to well-being, mental health and quality of life (Koening, 2004); however, religiosity and spirituality are not always associated with better health and well-being. Previous studies have reported that religious behavior is not beneficial for mental health, being associated rather with depressive symptoms (Van Herreweghe and Van Lancker, 2019) or being associated with depression for marital problems (Strawbridge et al., 1998), However, this study shows that for the Peruvian case, spirituality manifested in an individual, interior and subjective experience improves satisfaction with life.

In summary, as spirituality is an unidimensional theoretical construct (Fonseca Canteros, 2016), it has a direct relationship with life satisfaction, being an individual, inner, and subjective experience with a higher being (Rivera-Ledesma and Montero, 2005), understood as a human condition in which sense and purpose is looked for in a particular higher being (Fonseca Canteros, 2016). In this regard, Doolittle et al. (2013) found that those doctors who have an active spirituality were more protected against exhaustion.

Some limitations of this study should be pointed out. First, all data used for the study were taken from a sample that mainly included religious and Adventist people; thus, the possibility that some participants have given more denominational responses because of their religious conditions cannot be ruled out. Second, as participation was voluntary and virtual, some participants could be more motivated or inclined to report their experiences. Third, as it was a cross-sectional study, the behavior between variables was identified in a specific moment; therefore, other longitudinal studies are recommended.

Despite these limitations, this study has considerably widened the understanding of religiousness and spirituality in life satisfaction of Peruvian citizens during the COVID-19 pandemic.

Some strengths are also worth noting. First, the study makes a contribution to literature by approaching the variables “spirituality” and “religiousness”. Second, this study identified that spirituality predicts life satisfaction in Peruvian citizens thus providing great support for theoretical perspectives of spirituality and religiousness. Finally, those Peruvian citizens that showed higher levels of spirituality, also showed higher life satisfaction.

## Declarations

### Author contribution statement

Renzo Felipe Carranza Esteban, Josue Edison Turpo-Chaparro, Oscar Mamani-Benito: Conceived and designed the experiments; Performed the experiments; Analyzed and interpreted the data; Wrote the paper.

Jesús Hanco, Fiorella Sarria Arenaza: Contributed reagents, materials, analysis tools or data; Wrote the paper.

### Funding statement

This research did not receive any specific grant from funding agencies in the public, commercial, or not-for-profit sectors.

### Data availability statement

The data that has been used is confidential.

### Declaration of interests statement

The authors declare no conflict of interest.

### Additional information

No additional information is available for this paper.

## References

[bib1] Abbott R.P. (2020). ‘Providence’ or ‘religious fatalism’? A distinction without a difference in disasters research?. Prac. Theol..

[bib2] Ahmadi Z., Darabzadeh F., Nasiri M., Askari M. (2015). The effects of spirituality and religiosity on well-being of people with cancer: a literature review on current evidences. Jundishapur J. Chron. Dis. Care.

[bib3] Amaliah I., Aspiranti T., Purnamasari P. (2015). The impact of the values of islamic religiosity to islamic job satisfaction in tasikmalaya west Java, Indonesia. Ind. Cent. Proc. - Soc. Behav. Sci..

[bib65] Atienza F., Pons D., Balaguer I., García-Merita M. (2000). Propiedades psicométricas de la Escala de Satisfacción con la Vida en adolescentes. Psicothema.

[bib60] Ato M., López-García J.J., Benavente A. (2013). Un sistema de clasificación de los diseños de investigación en psicología. Anales de Psicología.

[bib4] Barreto P., Fombuena M., Diego R., Galiana L., Oliver A., Benito E. (2015). Bienestar emocional y espiritualidad al final de la vida. Med. Paliativa.

[bib61] BBC News Mundo https://www.bbc.com/mundo/noticias-52824767.

[bib5] Biccheri E., Roussiau N., Mambet-Doué C. (2016). Fibromyalgia, spirituality, coping and quality of life. J. Relig. Health.

[bib6] Bomhoff E.J., Siah A.K.L. (2019). The relationship between income, religiosity and health: their effects on life satisfaction. Pers. Indiv. Differ..

[bib7] Bovero A., Leombruni P., Miniotti M., Rocca G., Torta R. (2016). Spirituality, quality of life, psychological adjustment in terminal cancer patients in hospice. Eur. J. Canc. Care.

[bib8] Büssing A., Lötzke D., Glöckler M., Heusser P. (2015). Influence of spirituality on cool down reactions, work engagement, and life satisfaction in anthroposophic health care professionals. Evid. base Compl. Alternative Med..

[bib9] Castellanos-Torres E., Tomás Mateos J., Chilet-Rosell E. (2020). COVID-19 en clave de género. Gac. Sanit..

[bib72] Caycho-Rodríguez T. (2018). Tamaño del efecto para diferencia de medias: aportes complementarios. Enfermería Intensiva.

[bib73] Caycho-Rodríguez T. (2018). Importancia práctica de los resultados derivados de modelos de regresión: contribuciones a Madera-Anaya et al. Enfermería Clínica.

[bib74] Caycho-Rodríguez T. (2018). Tamaño del efecto en análisis de regresión en investigación geriátrica: comentarios a Rubio. Revista Española de Geriatría y Gerontología.

[bib10] Caycho-Rodríguez T., Ventura-León J., García Cadena C.H., Barboza-Palomino M., Arias Gallegos W.L., Dominguez-Vergara J., Samaniego Pinho A. (2018). Psychometric evidence of the diener’s satisfaction with life scale in peruvian elderly. Revista Ciencias de La Salud.

[bib68] Cohen J. (1988). Statistical power analysis for the behavioral sciences.

[bib62] Diener E., Emmons R.A., Larsen R.J., Griffin S. (1985). The satisfaction with life scale. Journal of Personality Assessment.

[bib11] Doolittle B.R., Windish D.M., Seelig C.B. (2013). Burnout, coping, and spirituality among internal medicine resident physicians. J. Grad. Med. Educ..

[bib12] Elgendy I.Y., Pepine C.J. (2020). Why are women better protected from COVID-19: clues for men? Sex and COVID-19. Int. J. Cardiol..

[bib13] Emmons R.A., Paloutzian R.F. (2003). The psychology of Religion. Annu. Rev. Psychol..

[bib14] Espinosa M.C. (2009). Ethnic spirituality, gender and health care in the Peruvian Amazon. Ethn. Health.

[bib15] Etemadifar S., Hosseiny R.S., Ziraki A., Omrani A., Alijanpoor M. (2016). The relationship between spiritual well-being and life satisfaction in females with infertility. Women’s Health Bull..

[bib16] Fardin M.A. (2020). COVID-19 epidemic and spirituality: a review of the benefits of religion in times of crisis. Jundishapur J. Chron. Dis. Care.

[bib64] Ferguson T. (2009). The ‘Write’ skills and more: A thesis writing group for doctoral students. Journal of Geography in Higher Education.

[bib75] Fetzer Institute y National Institute on Aging Working Group (1999). Multidimensional measurement of religiousness/spirituality for use in health research.

[bib17] Fonseca Canteros M. (2016). Importancia de los aspectos espirituales y religiosos en la atención de pacientes quirúrgicos. Rev. Chil. Cirugía.

[bib66] Gallardo-Peralta L.P., Cuadra-Peralta A., Veloso-Besio C. (2018). Validación de un índice Breve de Religiosidad y Espiritualidad en personas mayores. Revista de Psicología.

[bib18] Ghosh A., Arora B., Gupta R., Anoop S., Misra A. (2020). Effects of nationwide lockdown during COVID-19 epidemic on lifestyle and other medical issues of patients with type 2 diabetes in north India. Diab. Metab. Syndr.: Clin. Res. Rev..

[bib19] Gutiérrez M., Galiana L., Tomás J.M., Sancho P., Sanchís E. (2014). La predicción de la satisfacción con la vida en personas mayores de Angola: el efecto moderador del género. Psychosoc. Interv..

[bib20] Hill M.D. (2010). Myth, globalization, and mestizaje in new age andean religion: the intic churincuna (children of the sun) of urubamba, Peru. Ethnohistory.

[bib21] Jafari E., Dehshiri G.R., Eskandari H., Najafi M., Heshmati R., Hoseinifar J. (2010). Spiritual well-being and mental health in university students. Proc. Soc. Behav. Sci..

[bib22] Javanmard G.H. (2013). Religious beliefs and resilience in academic students. Proc. Soc. Behav. Sci..

[bib23] Johnstone B., Yoon D.P., Franklin K.L., Schopp L., Hinkebein J. (2009). Re-conceptualizing the factor structure of the brief multidimensional measure of religiousness/spirituality. J. Relig. Health.

[bib24] Kent B.V. (2020). Religion/spirituality and gender-differentiated trajectories of depressive symptoms age 13–34. J. Relig. Health.

[bib25] Kirkpatrick L.A. (1999). Toward an evolutionary psychology of religion and personality. J. Pers..

[bib26] Koenig H.G. (2004). Spirituality, wellness, and quality of life. Sex. Reproduction Menopause.

[bib27] Koenig H.G. (2020). Ways of protecting religious older adults from the consequences of COVID-19. Am. J. Geriatr. Psychiatr..

[bib29] Lavado Landeo L. (2018). Religiosidad de los médicos peruanos y su influencia en las decisiones bioéticas controversiales. Horizonte Médico (Lima).

[bib30] Lee S.B. (2011). P02-243 Life satisfaction, depression and spirituality for Korean elderly people. Eur. Psychiatr..

[bib31] Li S., Wang Y., Xue J., Zhao N., Zhu T. (2020). The impact of COVID-19 epidemic declaration on psychological consequences: a study on active weibo users. Int. J. Environ. Res. Publ. Health.

[bib32] Maier K., Surzykiewicz J. (2020). Mediated association between spirituality and life satisfaction in chronically ill undergraduate students. Psychol. Rel. Spiritual..

[bib33] Mejia C., Quispe-Sancho A., Rodriguez-Alarcon F., Ccasa-Valero L., Ponce-Lopez V., Sarela-Villanueva E., Vera-Gonzales J. (2020). Factores asociados al fatalismo ante la COVID-19 en 20 ciudades del Perú en marzo. Revista Habanera De Ciencias Medicas.

[bib34] Mirzaaghazadeh M., Farzan F., Amirnejad S., Hosseinzadeh M. (2016). Assessing the correlation of Machiavellian beliefs, spiritual intelligence and life satisfaction of Iran’s national team athletes (The Iranian national athletes as a Case Study). Pac. Sci Rev. B: Humanit. Soc. Sci..

[bib35] Moreira-Almeida A., Koenig H.G., Lucchetti G. (2014). Clinical implications of spirituality to mental health: review of evidence and practical guidelines. Rev. Bras. Psiquiatr..

[bib36] Park J., Roh S., Yeo Y. (2012). Religiosity, social support, and life satisfaction among elderly Korean immigrants. Gerontol..

[bib63] Pérez E.R., Medrano L. (2010). Análisis factorial exploratorio : Bases conceptuales y metodológicas. Revista Argentina de Ciencias Del Comportamiento.

[bib37] Plouffe R.A., Tremblay P.F. (2017). The relationship between income and life satisfaction: does religiosity play a role?. Pers. Indiv. Differ..

[bib38] Przepiorka A., Sobol-Kwapinska M. (2018). Religiosity moderates the relationship between time perspective and life satisfaction. Personality and Individual Differences.

[bib39] Puchalski C.M., Blatt B., Kogan M., Butler A. (2014). Spirituality and health: the development of a field. Acad. Med..

[bib40] Quadri S.A. (2020). COVID-19 and religious congregations: implications for spread of novel pathogens. Int. J. Infect. Dis..

[bib41] Rivera-Ledesma A., Montero M. (2005). Espiritualidad y religiosidad en adultos mayores Mexicanos. Salud Ment.

[bib42] Rodríguez-Ramos Á., Moriana J.A., García-Torres F., Ruiz-Rubio M. (2019). Emotional stability is associated with the MAOA promoter uVNTR polymorphism in women. Brain Behavi..

[bib43] Rożnowski B., Zarzycka B. (2020). Centrality of religiosity as a predictor of work orientation styles and work engagement: a moderating role of gender. Religions.

[bib44] Ruiz-Manriquez J., León-Lara X., Campos-Murguía A., Solis-Ortega A.A., Pérez-González B., Uscanga L.F., Peláez-Luna M. (2020). Conocimiento sobre la infección por SARS-CoV-2 de Gastroenterólogos y Endoscopistas de Latino América. Revista de Gastroenterología de México.

[bib45] Salmani S., Biderafsh A., Aliakbarzadeh Arani Z. (2020). The relationship between spiritual development and life satisfaction among students of qom university of medical sciences. J. Relig. Health.

[bib46] Sánchez-Lasheras M. (2016). Derecho y factor religioso en Chile y en el Perú. ¿Hacia la gestión pública de la diversidad religiosa?. Revista Chilena de Derecho.

[bib47] Schnettler B., Miranda-Zapata E., Grunert K.G., Lobos G., Denegri M., Hueche C., Poblete H. (2017). Life satisfaction of university students in relation to family and food in a developing country. Front. Psychol..

[bib48] Schnettler B., Miranda H., Sepúlveda J., Orellana L., Denegri M., Mora M., Lobos G. (2014). Variables que influyen en la satisfacción con la vida de personas de distinto nivel socioeconómico en el sur de Chile. Suma Psicol..

[bib49] Smith C. (2003). Theorizing religious effects among American adolescents. J. Sci. Stud. Relig..

[bib50] Strawbridge W.J., Shema S.J., Cohen R.D., Roberts R.E., Kaplan G.A. (1998). Religiosity buffers effects of some stressors on depression but exacerbates others. J. Gerontol. B Psychol. Sci. Soc. Sci..

[bib51] Urzúa A., Vera-Villarroel P., Caqueo-Urízar A., Polanco-Carrasco R. (2020). La Psicología en la prevención y manejo del COVID-19. Aportes desde la evidencia inicial. Ter. Psicol..

[bib52] Van Herreweghe L., Van Lancker W. (2019). Is religiousness really helpful to reduce depressive symptoms at old age? A longitudinal study. PloS One.

[bib53] Vang Z.M., Hou F., Elder K. (2019). Perceived religious discrimination, religiosity, and life satisfaction. J. Happiness Stud..

[bib54] Wildman W.J., Bulbulia J., Sosis R., Schjoedt U. (2020). Religion and the COVID-19 pandemic. Rel. Brain Behav..

[bib55] Wu R., Liu Z., Guo Q., Cai M., Zhou J. (2020). Couple similarity on personality, moral identity and spirituality predict life satisfaction of spouses and their offspring. J. Happiness Stud..

[bib56] Zadworna-Cieślak M. (2020). Spirituality, satisfaction with life and health-related behavior of older residents of long-term care institutions—a pilot study. Explore.

[bib57] Zavala M.W., Maliski S.L., Kwan L., Fink A., Litwin M.S. (2009). Spirituality and quality of life in low-income men with metastatic prostate cancer. Psycho Oncol..

[bib58] Zimmer Z., Jagger C., Chiu C.-T., Ofstedal M.B., Rojo F., Saito Y. (2016). Spirituality, religiosity, aging and health in global perspective: a review. SSM - Pop. Health.

